# Physicochemical, Functional, and Antioxidant Properties of Pectic Polysaccharides Extracted from Three Bast Fibrous Plants

**DOI:** 10.3390/life15101618

**Published:** 2025-10-16

**Authors:** Jialing Tang, Xi Li, Da Xu, Genggui Liu, Xiaoqin Zhang, Xiaofei Xiong, Xiai Yang, Xiaoli Qin, Yanchun Deng, Chunsheng Hou, Xiushi Yang

**Affiliations:** 1 Institute of Bast Fiber Crops, Chinese Academy of Agricultural Sciences, Changsha 410205, China; tangjialing@caas.cn (J.T.); 15831455718@163.com (D.X.); zhangxiaoqin@caas.cn (X.Z.); 15979912902@163.com (X.X.); yangxiai@caas.cn (X.Y.); qinxiaoli@caas.cn (X.Q.); dengyanchun@caas.cn (Y.D.); 2 Changsha Technology Innovation Center for Plant Bioactive Ingredient Identification and Biosynthesis, Changsha 410205, China; 3 Changsha Academy of Agricultural Sciences, Changsha 410026, China; lx443100@163.com; 4 School of Life Sciences, Inner Mongolia University, Hohhot 010021, China; 5 Changsha Huir Biological-Tech Co., Ltd., Changsha 410000, China; liugengui25@163.com

**Keywords:** bast fibrous plant, pectic polysaccharide, physicochemical characterization, functional property, antioxidant activity

## Abstract

Bast fibrous plants are recognized as potential sources of pectin, yet the structural characteristics and functional properties of pectic polysaccharides derived from these plants remain insufficiently investigated. In this study, three pectic polysaccharides (HP, KP, and RP) were isolated from the bast fibers of *Cannabis sativa* L. (hemp), *Hibiscus cannabinus* L. (kenaf), and *Boehmeria nivea* L. (ramie) using an ammonium oxalate solution. Their chemical composition, monosaccharide profile, molecular weight, microstructure, and functional groups were analyzed by spectroscopic and chromatographic techniques. These polysaccharides exhibited remarkable uronic acid content (50.49–61.14%), a relatively low degree of esterification (19.51–34.26%), and high molecular weights ranging from 147.10 to 242.16 kDa. The predominant neutral sugars identified were rhamnose, arabinose, and galactose. Furthermore, HP, KP, and RP demonstrated superior thermal stability, emulsifying properties, water/oil holding capacity, and cholesterol binding ability compared with commercial citrus pectin (CP). They also exhibited stronger radical scavenging activity, with KP showing particularly notable antioxidant performance (IC_50_ = 1856 and 1485 μg/mL for the DPPH and ABTS assays, respectively). Overall, these findings indicate that bast fibrous plants are promising alternative sources of pectic polysaccharides with favorable antioxidant properties, supporting their potential application as food additives or functional ingredients.

## 1. Introduction

Pectin is a complex heteropolysaccharides present in the middle lamella of the cell wall of higher plants. As a biopolymer, it is primarily composed of homogalacturonan, rhamnogalacturonan I, rhamnogalacturonan II, and xylogalacturonan domains [1]. With the advancement of the food industry, pectin is widely employed as a gelling agent, emulsifier, and stabilizer due to its ability to impart desirable texture and mechanical properties to food products. Moreover, as a dietary fiber, pectin exhibits various health benefits, such as antioxidant, anti-inflammatory, anticancer, and cardioprotective effects [2]. It has been utilized in the development of packaging films and coatings with antimicrobial and antioxidant properties for food preservation [3]. Pectin can be extracted using various methods with different solvents, including hot water, chelating agents, and acidic substances, assisted by enzymes, ultrasound, and microwave to enhance the extraction yield [4].

Although pectin is present in many plants, commercial production still relies predominantly on citrus peel, apple pomace, and sugar beet pulp [5]. The demand for pectin, both in food and pharmaceutical applications, continues to rise, with the global pectin market projected to reach a value of two billion USD by 2026 [6]. The limited supply from conventional sources, coupled with growing market demands, has spurred efforts to identify new pectin sources. Numerous studies have explored alternative materials for pectin extraction, such as broccoli stalks [7], burdock [8], cacao pod husk [9], and soy hull [10]. Nevertheless, there remains a strong need to identify pectin from non-traditional sources that possess functional properties comparable to those of commercial pectin.

Bast fibrous plants are economically important crops cultivated worldwide across Asia, Africa, Europe, America, and Oceania [11]. Among them, hemp (*Cannabis sativa* L.), kenaf (*Hibiscus cannabinus* L.), and ramie (*Boehmeria nivea* L.) are fast-growing species known for their high biomass yield and low input requirements. These plants are considered multipurpose resources, providing not only fibers but also raw materials for animal feed, food, and pharmaceuticals [12,13,14]. According to the Food and Agriculture Organization of the United Nations (FAO), the global production of hemp, kenaf, and ramie in 2023 reached 314,631, 227,908, and 9437 tons, respectively [15]. A typical bast fiber plant consists of bark, fiber bundles, and xylem. To isolate elementary fibers from the bundles, a degumming process is employed to separate them from the middle lamella, which is a gum-rich layer containing hemicellulose (5–21%), lignin (0.6–19%), and pectin (0.5–5%) [16,17]. Unfortunately, the degumming byproducts are often underutilized and discarded.

Hemp, kenaf, and ramie bast fibers have been identified as viable sources of pectin. Previous studies reported that pectic polysaccharides from retted hemp bast exhibit a high rhamnose-to-galacturonic acid ratio with very short neutral sugar side chains [18]. Pectin extracted from kenaf bast has shown good gel strength and a low degree of esterification [19,20]. Similarly, pectic fractions obtained from ramie degumming solutions have also been characterized as low in esterification [21]. However, the fine structural features and functional properties of pectic polysaccharides from these bast fibrous plants remain inadequately studied, and comparative analyses among them are scarce.

Therefore, the aim of this study was to characterize the structural and functional properties of pectic polysaccharides extracted from hemp, kenaf, and ramie bast fibers and to evaluate their antioxidant activities. The results are expected to deepen the understanding of pectin derived from bast fibers and support their potential application in the food industry.

## 2. Materials and Methods

### 2.1. Materials

The bast fibers of hemp (“Yunma-7” var.), kenaf (“Zhonghongma-13” var.), and ramie (“Zhongzhuma-1” var.) were collected from the experimental fields of the Institute of Bast Fiber Crops (IBFC), Chinese Academy of Agricultural Sciences (Changsha, China). Details regarding the sample images and origins are provided in Table 1. The samples were provided and botanically identified by Professor Shengwen Duan from IBFC. The bast was mechanically separated from the core of well-grown mature plants and subsequently air-dried. Commercial citrus pectin (CP) was purchased from Shanghai Aladdin Bio-Chem Technology Co., Ltd. (Shanghai, China). Standard monosaccharides, including fucose (Fuc), arabinose (Ara), rhamnose (Rha), galactose (Gal), glucose (Glc), xylose (Xyl), mannose (Man), fructose (Fru), galacturonic acid (GalA), and glucuronic acid (GlcA), were acquired from Sigma-Aldrich (St. Louis, MO, USA). The 1,1-diphenyl-2-picrylhydrazyl (DPPH) and 2,2′-azinobis (3-ethylbenzothiazoline-6-sulfonic acid) ammonium salt (ABTS) were supplied by Solarbio Life Sciences (Beijing, China). All other chemical reagents, such as ethanol, ammonium oxalate, sodium hydroxide, and hydrochloric acid, were of analytical grade and purchased from Shanghai Macklin Biochemical Technology Co., Ltd. (Shanghai, China).

### 2.2. Extraction of Pectic Polysaccharides

Pectic polysaccharides were extracted from the bast fibers using previously described methods with minor modifications [19,22]. Briefly, bast fibers from hemp, kenaf, and ramie were cut into 1–2 cm pieces with a scissor and then crushed in a blender (L18 Y916, Joyoung Company Ltd., Jinan, China) after drying at 40 °C in an oven (101-2AB, Beijing Zhongxingweiye Company Ltd., Beijing, China). The powder was first refluxed with 95% ethanol for 3 h to obtain alcohol-insoluble residues, which were collected and dried at 40 °C. Pectic polysaccharides were then extracted by treating the residue with 4 g/L ammonium oxalate solution in a boiling water bath (DZKW-4, Beijing Zhongxingweiye Company Ltd., Beijing, China) for 3 h at a liquid-to-solid ratio of 15:1 (*v*/*w*). After cooling to room temperature, the mixture was centrifuged at 4000 rpm for 20 min. The supernatant was collected and adjusted to pH 4.5 with diluted hydrochloric acid. The solution was precipitated with precooled absolute ethanol (1:4, *v*/*v*). The mixture was kept at 4 °C overnight before filtration. Lastly, the resulting precipitate was washed three times with 95% ethanol, resuspended in deionized water, and dialyzed (molecular weight cut-off: 6.0–8.0 kDa) against deionized water for 48 h, with water changes every 12 h. Finally, the retentate was freeze-dried, ground into powder, and stored at 4 °C for further analysis.

### 2.3. Determination of Physicochemical Properties

#### 2.3.1. Chemical Composition Analysis

The total carbohydrate content was measured using the phenol-sulfuric acid method with calibration factors according to our previous report [23]. Uronic acid content was quantified using the m-hydroxybiphenyl method, with D-galacturonic acid as the standard, by measuring absorbance at 520 nm [24]. Protein content was determined using the Bradford method, with bovine serum albumin as the standard, and measured by absorbance at 595 nm [25]. The total polyphenol content was determined using the Folin–Ciocalteu colorimetric method, with gallic acid as the standard, and absorbance was measured at 760 nm [26]. The degree of esterification was determined according to the sodium hydroxide titration method outlined in the Food Chemical Codex [27].

#### 2.3.2. Monosaccharide Composition Analysis

Monosaccharide composition of polysaccharides was analyzed by ion chromatography [28], which was a type of liquid chromatography specifically designed for the analysis of anions and cations in various samples. Briefly, 5 mg of each sample was hydrolyzed with 10 mL of 2 M trifluoroacetic acid (TFA) at 121 °C for 2 h. The hydrolysate was evaporated to dryness under a nitrogen stream, and residual TFA was removed by washing three times with methanol. The dried residue was dissolved in deionized water and filtered through a 0.22 μm membrane. Analysis was performed using an ion chromatography system (Dionex ICS 5000+, Thermo Fisher Scientific, Carlsbad, CA, USA) equipped with a CarboPac PA-20 IC column (150 mm × 3.0 mm, 10 μm; Thermo Scientific). The mobile phase consisted of (A) H_2_O, (B) 0.1 M NaOH, and (C) 0.1 M NaOH containing 0.2 M sodium acetate. Gradient elution was applied at a flow rate of 0.5 mL/min, and the column temperature was maintained at 30 °C. Quantification was performed using external standard calibration curves.

#### 2.3.3. Molecular Weight Distribution

The weight-average molecular weight (*M*_w_) and number-average molecular weight (*M*_n_) were determined using a multi-angle laser light scattering system (DAWN HELEOS-II, Wyatt Technology Co., Santa Barbara, CA, USA) coupled with an Optilab T-rEX differential refractive index detector (Wyatt Technology Co.), following a previously reported method [29]. Separation was performed using three tandem columns (Shodex OH-pak SB-805, 804, and 803; 300 × 8 mm; Showa Denko K.K., Tokyo, Japan) maintained at 45 °C. Samples were dissolved in the mobile phase of 0.1 M NaNO_3_ aqueous solution containing 0.02% NaN_3_ at a concentration of 1 mg/mL and filtered through a 0.45 μm membrane. The mobile phase flow rate was 0.3 mL/min. The refractive index increment (*dn*/*dc*) was determined as 0.141 mL/g

#### 2.3.4. Scanning Electron Microscopy (SEM)

The surface morphology of the polysaccharides was observed using a scanning electron microscope (TESCAN MIRA LMS, TESCAN ORSAY HOLDING, Brno-Kohoutovice, Czech Republic). Lyophilized samples were mounted on a specimen holder and coated with platinum following a previously described procedure [29]. Imaging was performed at an accelerating voltage of 5 keV with magnifications of 500× and 10,000×.

#### 2.3.5. Fourier Transform Infrared Spectroscopy (FT-IR)

Freeze-dried pectic samples were mixed with KBr (1:100, *w*/*w*) and pressed into pellets [30]. FT-IR spectra were recorded on an IRSpirit spectrometer (Shimadzu Corporation, Kyoto, Japan) in the range of 400–4000 cm^−1^ at a resolution of 4 cm^−1^.

### 2.4. Determination of Functional Properties

#### 2.4.1. Thermal Analysis

Thermogravimetry analysis was conducted using a Mettler TGA 2 thermal analyzer (Mettler-Toledo (Schweiz) GmbH, Greifensee, Switzerland) according to a previously described method with minor modification [31]. Briefly, samples were placed in an alumina crucible and heated from 30 to 400 °C at a rate of 10 °C/min under a nitrogen atmosphere. An empty aluminum pan was used as a reference.

#### 2.4.2. Emulsifying Properties Evaluation

Emulsifying capacity (EC) and emulsion stability (ES) were evaluated according to the method described by Bayar et al. [32] with slight modifications. Briefly, polysaccharide dispersions (2% and 4%, *w*/*v*) were mixed with corn oil (10 mL sample + 5 mL oil) and homogenized at 8000 rpm for 3 min. The emulsion was centrifuged at 1154× *g* for 5 min, and EC was calculated as the ratio of emulsified layer volume to total mixture volume. For ES, emulsions were heated at 80 °C for 30 min, and then centrifuged at 104× *g* for 5 min. ES was calculated as the ratio of the remaining emulsion volume divided by the initial emulsified layer volume.

#### 2.4.3. Rheological Behaviors

Steady shear viscosity of polysaccharide solutions (2.0% and 4.0%, *w*/*w*) was measured using an Anton Paar MCR 92 rheometer (Anton Paar GmbH, Graz, Austria) with a cone and plane geometry system (cone diameter, 50 mm; angle, 1°) [33]. After equilibration for 5 min, the steady-state flow test was performed with the shear rate increased from 0.1 to 200 s^−1^ at 25 °C.

#### 2.4.4. Adsorption Ability Determination

Water retention capacity (WRC) was determined as described previously [34]. Samples were mixed with deionized water (1:10, *w*/*v*), vortexed, and centrifuged at 3000× *g* for 15 min. WRC was expressed as grams of water held per gram of sample.

Oil holding capacity (OHC) was measured according to the method described by Muñoz-Almagro et al. [35]. Samples were suspended in corn oil (1:20, *w*/*v*), stirred for 30 min, and centrifuged at 3000× *g* for 30 min. OHC was calculated as grams of oil retained per gram of sample.

Cholesterol binding capacity (CBC) was determined with slight modifications to the reported methods [31,36]. Briefly, samples were incubated with cholesterol solution (1 mg/mL, in ethanol) at 37 °C for 60 min with stirring, and then filtered. The filtrate (0.3 mL) was mixed with acetic acid (0.2 mL) and o-phthaldialdehyde (1.5 mL), followed by adding 4 mL of sulfuric acid-acetic acid mixture (50:50, *v*/*v*). After 10 min, absorbance was measured at 550 nm. A cholesterol standard curve was used for quantification, and CBC was expressed as mg cholesterol adsorbed per gram of sample.

### 2.5. Measurement of Antioxidant Activity

DPPH and ABTS radical scavenging assays were performed as described previously [37]. Briefly, a series of polysaccharide solution (200, 400, 800, 1600, and 3200 μg/mL) was prepared. The solution was mixed with 0.5 mL of 0.004% (*w*/*v*) DPPH solution (in anhydrous ethanol) and incubated in the dark at 35 °C for 30 min. Absorbance was measured at 517 nm. For the ABTS assay, the sample solution was mixed with 1 mL of ABTS working solution, and absorbance was read at 734 nm. Ascorbic acid (6, 12, 24, 48, and 96 μg/mL) was used as a positive control for both assays.

### 2.6. Statistical Analysis

All experiments were performed in triplicate. Data are presented as mean ± standard deviation. Differences among groups were assessed using Duncan’s multiple range tests in SPSS 20.0 software (SPSS Inc., Chicago, IL, USA), with statistical significance set at *p <* 0.05.

## 3. Results and Discussion

### 3.1. Extraction Yield

The extraction yields of pectic polysaccharides from hemp (HP), kenaf (KP), and ramie (RP) ranged from 7.70% to 11.74% (Table 2). HP showed the highest yield, which is consistent with a previously reported extraction rate for hemp pectin (10.46%) [19]. Notably, the yield of KP was considerably higher than the 4–5% reported in earlier studies [38]. The yields of HP and KP also exceeded that of pectin conventionally extracted from cacao pod husk (8.3%) [9]. For ramie, the literature values for pectin extraction yield vary widely, from as low as 1.11% [21] to as high as 15.81% [22], indicating that our result falls within this broad range. However, the yields of these pectic polysaccharides from the three bast fibers (BFPs) were all lower than that of citrus peel pectin (21.85%) [39], one of the primary commercial sources of pectin.

### 3.2. Physicochemical Properties

#### 3.2.1. Chemical Compositions

Generally, the chemical composition of pectin exhibits variation across different sources. As summarized in Table 2, the three pectic polysaccharides exhibited distinct contents of uronic acid (UA), protein, total polyphenol (TP), and degree of esterification (DE). These compositional differences may contribute to variations in their functional properties and bioactivities.

Galacturonic acid content and degree of esterification are critical parameters for assessing pectin quality [2]. Among the three pectic polysaccharides, UA and DE values ranged from 50.49% to 61.14% and 19.51% to 34.26%, respectively. HP exhibited the highest UA content, which is comparable to the galacturonic acid level (63%) of oxalate-extractable pectic polysaccharide from hemp fiber bundles [18]. The UA content of RP (54.63%) was slightly higher than the value (49.92%) previously determined for ramie pectin using the carbazole colorimetry method [22]. RP showed the highest DE, followed by KP and HP. However, these DE values were lower than those reported for pectin from ramie (53.11%) [22] and green kenaf bast (46–48%) [38]. Consistent with our findings, pectin extracts from hemp have been previously described as partially esterified, containing 2–5% acetyl groups and less than 1% methyl esters [18].

Protein and phenolic compounds were detected in all three pectic polysaccharides. RP had the highest protein content (5.93%), while KP contained the most abundant polyphenols (21.77 mg GAE/g). Previous studies have reported the presence of protein and phenolics in the phloem of hemp, kenaf, and ramie [38,40,41]. These components may form complexes with pectin and thus be co-extracted by ammonium oxalate. The presence of protein and phenolics is likely to enhance the functional and bioactive potential of the polysaccharides.

#### 3.2.2. Monosaccharide Composition

Nine monosaccharides were detected in the pectic samples by ion chromatography analysis. As shown in Table 3, the proportions of most monosaccharides differed significantly among the three polysaccharides. Rhamnose, arabinose, galactose, glucose, xylose, mannose, and galacturonic acid were previously identified in oxalate-extractable pectic polysaccharides from hemp bast fibers [18], though fucose and glucuronic acid were not reported in that study. In contrast, glucuronic acid has been detected in ramie leaf polysaccharides extracted using a deep eutectic solvent with microwave assistance [42]. Interestingly, pectin extracted from raw ramie fiber using citric acid exhibited a simpler monosaccharide profile, consisting only of rhamnose, galactose, arabinose, and galacturonic acid [17]. These comparisons suggest that the extraction solvent significantly influences the monosaccharide composition of the resulting pectic polysaccharides.

Rhamnose, arabinose, and galactose were the main neutral sugars in all three pectic samples, accounting for 73.22% to 83.65% of the total monosaccharides. RP and KP showed the highest proportions of rhamnose (41.69%) and arabinose (38.46%), respectively. Rhamnose is a major component of the rhamnogalacturonan I (RG-I) backbone, while arabinose predominantly constitutes the side chains of RG-I [43]. These results suggest that the three pectic polysaccharides might possess a branched RG-I structure, which aligns with the structural features reported for hemp pectic polysaccharides [18] and ramie pectin [17].

#### 3.2.3. Molecular Weight Analysis

**Figure 1:**
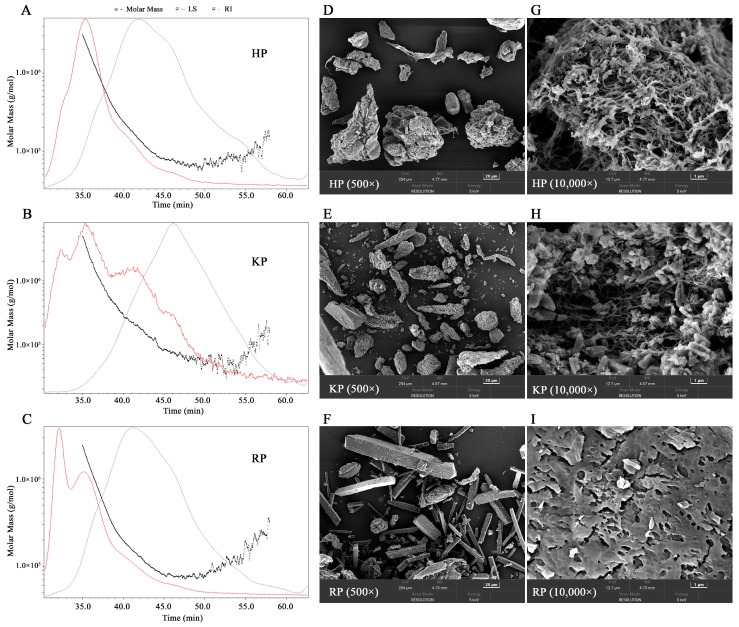
Chromatograms of the molar mass distribution (**A**–**C**) and scanning electron microscope (SEM) spectrum (**D**–**I**) of pectic polysaccharides from bast fibers of hemp (HP), kenaf (KP), and ramie (RP). For molar mass distribution analysis, the light scattering (LS) and refractive index (RI) signals are detected. For SEM analysis, images with magnification ratios of 500 and 10,000 times are shown.

The molecular weight distributions of the pectic samples were determined by size exclusion chromatography coupled with multi-angle laser light scattering and refractive index detection (SEC-MALLS-RI). As shown in Figure 1A–C, the refractive index (RI) signals displayed symmetrical single peaks for all polysaccharides. The *M*_w_ was highest in RP (242.16 kDa), followed by HP (232.25 kDa) and KP (147.10 kDa), as summarized in Table 3. These *M*_w_ values are higher than those reported for ramie leaf polysaccharides (23.4 kDa) extracted using deep eutectic solvent [42]. In comparison, one of them (KP) is slightly lower than the *M*_w_ of citrus pectin (154.7 kDa) obtained via hot water maceration [44]. The polydispersity index (PDI), calculated as *M*_w_/*M*_n_, was used to evaluate the uniformity of the molecular weight distribution [10]. The PDI of RP was higher than that of HP and KP, indicating a broader molecular weight distribution in RP.

#### 3.2.4. SEM Analysis

The surface morphologies of pectic samples were examined using scanning electron microscopy. As shown in Figure 1D–I, at 500× magnification, HP and KP exhibited irregular block and strip-like shapes, whereas RP displayed numerous rectangular blocks. At 10,000× magnification, RP showed a lamella structure with numerous scattered pores on the surface. In contrast, the magnified images of HP and KP revealed filamentous network structures containing multiple pores, which may contribute to their adsorption potential [31]. The microstructure differences among the pectic samples are likely attributable to variations in their physicochemical properties, such as monosaccharide composition and molecular weight, as well as the random coagulation of polysaccharides during ethanol precipitation [10,29].

#### 3.2.5. FT-IR Analysis

**Figure 2:**
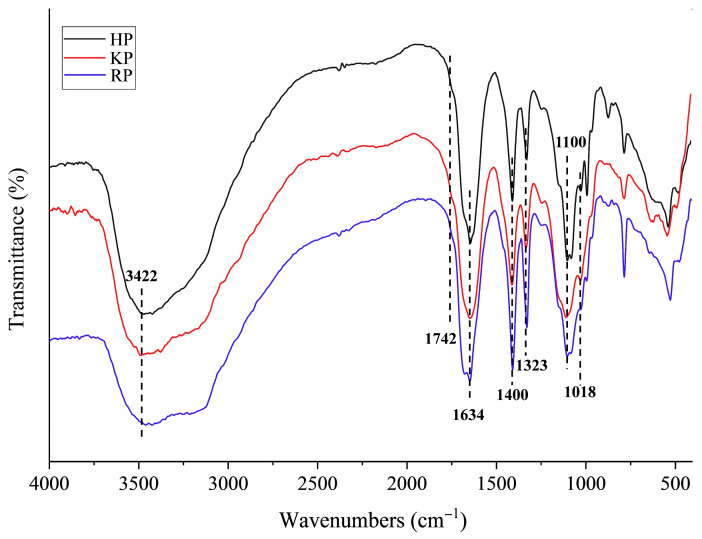
Fourier transform infrared spectroscopy (FT-IR) spectrum of pectic polysaccharides from bast fibers of hemp (HP), kenaf (KP), and ramie (RP).

FT-IR analysis was conducted, and the resulting spectra are presented in Figure 2. The three polysaccharides showed similar spectral profiles, with characteristic absorption peaks corresponding to key structural features. A broad and prominent band around 3422 cm^−1^, attributed to O–H stretching vibrations of intramolecular hydroxyl groups [45], was observed in all spectra. A weak band near 1742 cm^−1^ was associated with the C=O stretching vibration of esterified carboxyl groups [37]. The signals around 1634 cm^−1^ in all samples were ascribed to asymmetric stretching vibrations of free carboxylate groups [46]. Consistent with previously reported FT-IR spectra of pectin from raw hemp [19], the strong absorption bands between 1634 cm^−1^ and 1400 cm^−1^ were assigned to O–H deformation vibrations of carboxylic acid, while those between 1400 cm^−1^ and 1323 cm^−1^ likely correspond to saturated C–H bending vibrations. These characteristic bands are also in agreement with previously reported FT-IR spectra of pectin from ramie fiber [17]. Additionally, the peak at 1100 cm^−1^ represents vibrations of C–OH side groups, and the peak at 1018 cm^−1^ is characteristic of D-pyranose sugar [47]. Collectively, these spectral features confirm that the samples are pectic polysaccharides.

### 3.3. Functional Properties

#### 3.3.1. Thermal Stabilities

**Figure 3:**
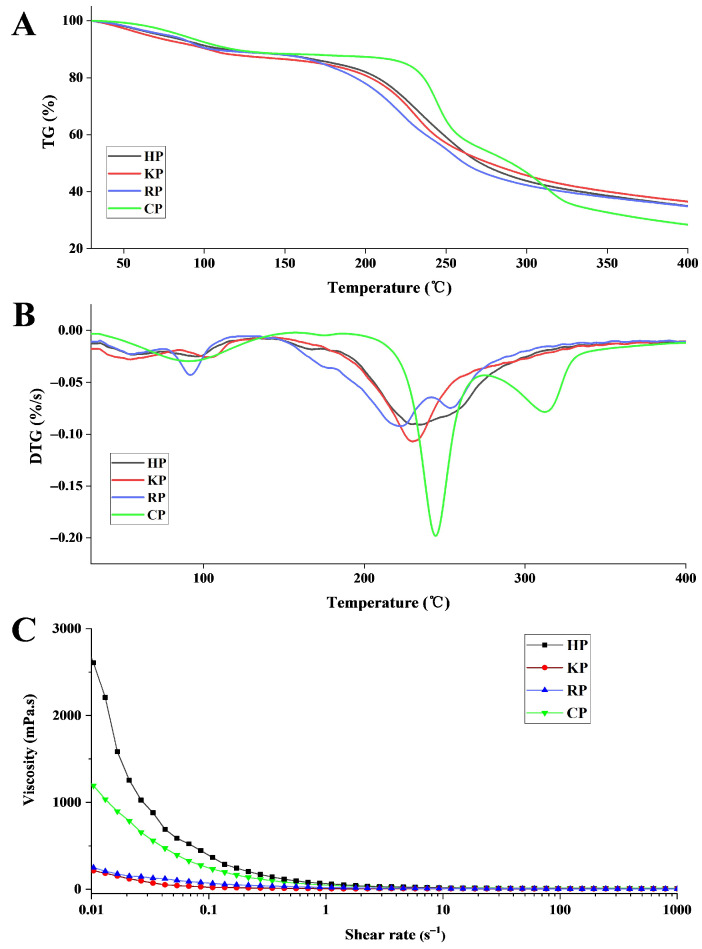
TG (**A**), DTG (**B**), and viscosity (**C**) curves of pectic polysaccharides from bast fibers of hemp (HP), kenaf (KP), ramie (RP), and commercial pectin (CP). TG, thermogravimetry; DTG, derivative thermogravimetry.

The thermal stabilities of the pectic polysaccharides were evaluated by thermogravimetry (TG) and derivative thermogravimetry (DTG). The TG curve records the change in sample weight during heating, while the DTG curve reflects the rate of degradation. For comparative purposes, a commercial citrus pectin (CP) was included in the analysis. As shown in Figure 3A,B, the TG/DTG curves of HP and KP exhibited similar trends, which could be divided into three main stages. In the first stage (30–180 °C), a slight weight loss occurred, primarily due to the evaporation of moisture and volatile components [31]. During the second stage (180–330 °C), a rapid decrease in weight was observed, attributed to the pyrolysis and thermal decomposition of the polysaccharide backbone [37]. This stage may also involve the formation of gaseous products and solid char. In the final stage (330–400 °C), weight loss proceeded at a slower rate, likely resulting from the further decomposition of the char residue [30].

The DTG curves reveal a single weight loss peak in the second stage for KP (230.47 °C) and HP (235.73 °C), whereas RP (222.13 °C and 253.68 °C) and CP (244.65 °C and 312.81 °C) showed two distinct peaks. After the entire heating process, the residual weight percentages of KP, HP, RP, and CP were 36.53%, 34.99%, 34.85%, and 28.42%, respectively. These results suggest that the three pectic polysaccharides possess comparable thermal stability, which was higher than that of CP across the tested temperature range.

#### 3.3.2. Emulsifying Properties

The emulsifying capacity (EC) and emulsifying stability (ES) of the pectic samples are summarized in Table 4. EC and ES values followed the order of KP > RP > HP > CP and KP > HP > RP > CP, respectively, indicating that KP exhibited the best emulsifying performance. All three pectic polysaccharides demonstrated superior emulsifying ability compared to CP. At a concentration of 2%, the EC values of pectic polysaccharides (34.38–41.25%) were higher than those reported for water-soluble pectin from Chinese dwarf cherry fruits (31.35%) and pectin from *Opuntia ficus indica* cladodes (19.23%) [31,32]. The emulsifying activity of pectin is influenced by the protein content, degree of methyl esterification, and the presence of neutral sugar side chains, whereas emulsifying stability is attributed to steric stabilization by a hydrated protein layer and electrostatic interactions of carboxylic acid groups [48]. Additionally, phenolic compounds have been reported to affect the emulsion performance of pectin [49]. In this study, the three pectic polysaccharides contained notable amounts of protein and polyphenols, as well as arabinose side chains. These structural characteristics likely contribute to their enhanced emulsifying properties compared to commercial pectin.

#### 3.3.3. Rheological Property

The rheological behavior of the polysaccharide solutions was assessed by measuring steady shear viscosity. As shown in Figure 3C, the apparent viscosity of all four samples decreased rapidly in the low shear rate region and then declined more gradually at higher shear rates. HP exhibited the highest viscosity at the same shear rate, followed by CP. And they both demonstrated pronounced shear-thinning behavior, a characteristic of non-Newtonian fluids. In contrast, KP and RP showed lower viscosity values and similar flow curves. It was proposed that polysaccharides with higher *M*_w_ tend to impede fluid motion more effectively, leading to higher viscosity [50]. Moreover, pectin viscosity is also enhanced by a higher degree of esterification, protein content, polydispersity index, and branching degree [51]. Therefore, the high viscosity of HP may be comprehensively attributed to its relatively high *M*_w_, protein content, and arabinose side chains. These rheological properties suggest that HP has promising potential as a natural thickener in the food industry.

#### 3.3.4. Adsorption Ability

The water retention capacity (WRC) reflects the amount of water bound by pectic samples and is an important parameter influencing the drying behavior, texture, and bulk volume of food matrices [52]. As shown in Table 4, KP showed the highest WRC, followed by HP and RP. The WRC values of these samples (4.17–8.19 g/g) were all higher than that of CP (2.39 g/g). WRC is known to be affected by molecular weight, galacturonic acid, and particle size [51]. The favorable WRC of KP, HP, and RP suggests their application potential in preventing syneresis in food products.

The oiling holding capacity (OHC) indicates the amount of oil adsorbed by the pectic samples and is a key functional property of pectic hydrocolloids. It reflects the ability to stabilize oil-rich emulsions and reduce oil absorption during digestion [35]. Notably, the OHC values of the three pectic polysaccharides were substantially higher than that of CP (1.36 g/g). KP exhibited the highest OHC (10.27 g/g), followed by HP (7.54 g/g) and RP (5.39 g/g). OHC is influenced by structural features such as polysaccharide hydrophilicity and branching degree [52]. The superior OHC of KP may be attributed to its higher amount of arabinose side chains.

The cholesterol binding capacity (CBC) is used to evaluate the ability of a polysaccharide to adsorb food-derived cholesterol. The CBC values of pectic samples followed the order: KP > HP > RP > CP. This capacity is associated with the presence of hydrophobic groups and side chains in the polysaccharide structure [31]. KP, which had the highest arabinose content and an interconnected filamentous network structure, showed the highest CBC. Overall, the three pectic polysaccharides exhibited better cholesterol binding ability than citrus pectin, indicating their potential use in healthy foods aimed at reducing cholesterol adsorption.

### 3.4. Antioxidant Activity

**Figure 4:**
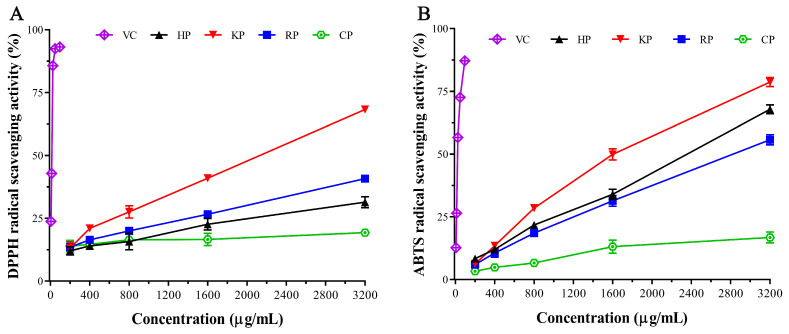
DPPH (**A**) and ABTS (**B**) radical scavenging activity of pectic polysaccharides from bast fibers of hemp (HP), kenaf (KP), and ramie (RP), ascorbic acid (VC), and commercial pectin (CP).

The antioxidant activity of the pectic polysaccharides was evaluated using two common assays: DPPH radical scavenging, which measures the ability of hydrogen-donating antioxidants to reduce the stable DPPH radical, and ABTS radical cation scavenging, based on electron transfer to form a stable colored product [23]. As shown in Figure 4A,B, both DPPH and ABTS scavenging activities increased with increasing polysaccharide concentration (200–3200 μg/mL). The three pectic polysaccharides exhibited stronger scavenging capacity than CP at most concentrations, though lower than that of ascorbic acid (VC). Among the polysaccharides, KP showed the highest DPPH scavenging activity, with the lowest IC_50_ value (1856 μg/mL), followed by RP (IC_50_ = 7288 μg/mL) and HP (IC_50_ = 18,364 μg/mL). Similarly, KP displayed the strongest ABTS scavenging activity (IC_50_ = 1485 μg/mL), while HP (IC_50_ = 2134 μg/mL) was more effective than RP (IC_50_ = 2796 μg/mL). The scavenging activities of KP and RP were higher than those reported for pectins from *Opuntia ficus indica* cladodes [32] and Algerian dates (*Phoenix dactylifera* L.) [53]. These results indicate that all three pectic polysaccharides possess strong radical scavenging abilities.

The antioxidant activity of pectin is closely related to its chemical composition and structural features. It should be noted that the three pectic polysaccharides were not purified and contained considerable protein (2.46–5.93%) and phenolics (7.12–21.77 mg GAE/g), both of which are reported to positively correlate with scavenging activity [54,55]. In addition, monosaccharide composition, uronic acid content, and molecular weight also contribute significantly to the antioxidant properties of polysaccharides [56]. Therefore, the antioxidant activities observed in this study are likely the result of synergistic effects among these compositional and structural factors.

## 4. Conclusions

In summary, three pectic fractions were successfully isolated from the bast fibers of hemp, kenaf, and ramie. Their structural characteristics, functional properties, and antioxidant activities were systematically investigated. Structural analysis indicated that all three are low-esterified pectic polysaccharides, rich in arabinose, rhamnose, and galactose, with relatively high molecular weights. These pectic fractions exhibited favorable thermal stability, emulsifying capacity, water retention capacity, oil-holding capacity, viscosity, and cholesterol-binding ability. The superior thermal stability and emulsifying performance of pectic polysaccharides from kenaf may be attributed to cross-linking between polysaccharides, proteins, and polyphenols. Its outstanding cholesterol-binding capacity likely arises from its distinct arabinose content and filamentous network structure. The strong radical scavenging activity observed, particularly that of the pectic polysaccharide from kenaf, could be largely attributed to its high polyphenol content. Overall, these findings underscore the potential of bast fiber plants as valuable sources of pectic polysaccharides for use as novel emulsifiers, thickeners, and antioxidants in the food industry.

## Figures and Tables

**Table 1:** Sample information on hemp, kenaf, and ramie.

Sample	Hemp	Kenaf	Ramie
Image of plant and bast	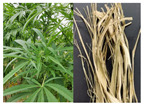	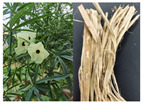	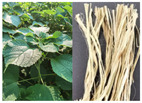
Scientific name	*Cannabis sativa* L.	*Hibiscus cannabinus* L.	*Boehmeria nivea* L.
Origin	Chuxiong Prefecture, Yunnan Province, China	Yiyang City, Hunan Province, China	Yiyang City, Hunan Province, China
Abbreviation of extracted polysaccharides	HP	KP	RP

**Table 2:** Physicochemical properties of pectic polysaccharides from bast fibers of hemp (HP), kenaf (KP), and ramie (RP).

	HP	KP	RP
Yield (%)	11.74 ± 0.25 a	8.68 ± 0.14 b	7.70 ± 0.31 c
Uronic acid (%)	61.14 ± 0.58 a	50.49 ± 0.80 c	54.63 ± 0.84 b
DE (%)	19.51 ± 1.75 c	24.43 ± 1.36 b	34.26 ± 1.89 a
Protein (%)	4.82 ± 0.06 b	2.46 ± 0.02 c	5.93 ± 0.07 a
TP (mg GAE/g)	7.12 ± 0.05 c	21.77 ± 0.44 a	9.85 ± 0.10 b

Note: DE, degree of esterification; TP, total polyphenol; GAE, gallic acid equivalents. Values are presented as mean ± SD, with different letters in the same row representing significant differences at *p* < 0.05.

**Table 3:** Monosaccharide composition and molecular weight of pectic polysaccharides from bast fibers of hemp (HP), kenaf (KP), and ramie (RP).

	HP	KP	RP
Monosaccharides (%)			
Fuc	1.40 ± 0.01 a	0.81 ± 0.02 c	1.25 ± 0.03 b
Rha	23.64 ± 0.06 b	18.84 ± 0.14 c	41.69 ± 0.28 a
Ara	26.13 ± 0.17 b	38.46 ± 0.03 a	15.16 ± 0.15 c
Gal	23.45 ± 0.11 b	26.35 ± 0.21 a	26.23 ± 0.18 a
Glc	5.68 ± 0.02 a	3.29 ± 0.03 c	4.36 ± 0.01 b
Xyl	4.16 ± 0.04 a	2.17 ± 0.05 b	2.33 ± 0.04 b
Man	3.88 ± 0.01 a	3.28 ± 0.01 b	2.64 ± 0.02 c
GalA	9.87 ± 0.08 a	5.04 ± 0.05 b	4.83 ± 0.03 b
GlcA	1.79 ± 0.02 a	1.76 ± 0.01 a	1.51 ± 0.02 b
Molecular weight			
*M*_w_ (kDa)	232.25 ± 4.08 b	147.10 ± 3.32 c	242.16 ± 6.64 a
*M*_n_ (kDa)	121.48 ± 2.63 a	78.10 ± 1.52 c	109.33 ± 1.78 b
Polydispersity index	1.91 ± 0.06 b	1.88 ± 0.07 b	2.22 ± 0.05 a

Note: Values are presented as mean ± SD, with different letters in the same row representing significant differences at *p* < 0.05.

**Table 4:** The emulsifying property and adsorption capacity of pectic polysaccharides from bast fibers of hemp (HP), kenaf (KP), ramie (RP), and commercial pectin (CP).

Parameters	Concentration	HP	KP	RP	CP
EC (%)	2%	34.38 ± 0.56 c	41.25 ± 1.76 a	35.21 ± 1.24 b	33.70 ± 0.71 c
	4%	37.75 ± 1.11 c	43.76 ± 1.87 a	40.23 ± 0.93 b	36.88 ± 0.88 d
ES (%)	2%	62.50 ± 1.79 a	63.67 ± 0.65 a	57.69 ± 1.12 b	55.56 ±1.31 c
	4%	81.70 ± 1.49 b	82.92 ± 1.35 a	79.69 ± 2.21 c	67.82 ± 1.63 d
WRC (g/g)	/	6.90 ± 0.11 b	8.19 ± 0.10 a	4.17 ± 0.07 c	2.39 ± 0.10 d
OHC (g/g)	/	7.54 ± 0.48 b	10.27 ± 0.32 a	5.39 ± 0.24 c	1.36 ± 0.11 d
CBC (mg/g)	/	9.06 ± 0.41 b	12.17 ± 0.51 a	7.14 ± 0.30 c	3.04 ± 0.21 d

Note: EC, emulsifying capacity; ES, emulsion stability; WRC, water retention capacity; OHC, oil holding capacity; CBC, cholesterol binding capacity. Values are presented as mean ± SD, with different letters in the same row representing significant differences at *p* < 0.05.

## Data Availability

The raw data supporting the conclusions of this article will be made available by the authors on request.

## References

[1] Kalita P., Rahman M.T., Haloi P., Bora N.S., Pachuau L. (2025). Pectin in Gut Health and beyond: A Review. Int. J. Biol. Macromol..

[2] Barrera-Chamorro L., Fernandez-Prior Á., Rivero-Pino F., Montserrat-de La Paz S. (2025). A Comprehensive Review on the Functionality and Biological Relevance of Pectin and the Use in the Food Industry. Carbohydr. Polym..

[3] Syarifuddin A., Muflih M.H., Izzah N., Fadillah U., Ainani A.F., Dirpan A. (2025). Pectin-Based Edible Films and Coatings: From Extraction to Application on Food Packaging towards Circular Economy—A Review. Carbohydr. Polym. Technol. Appl..

[4] Dixit S.S., Muruganandam L., Ganesh Moorthy I. (2025). Pectin from Fruit Peel: A Comprehensive Review on Various Extraction Approaches and Their Potential Applications in Pharmaceutical and Food Industries. Carbohydr. Polym. Technol. Appl..

[5] Peng J., Bu Z., Ren H., He Q., Yu Y., Xu Y., Wu J., Cheng L., Li L. (2022). Physicochemical, Structural, and Functional Properties of Wampee (*Clausena lansium* (Lour.) Skeels) Fruit Peel Pectin Extracted with Different Organic Acids. Food Chem..

[6] Jia Y., Wang C., Khalifa I., Zhu Y., Wang Z., Chen H., Liang X., Zhang H., Hu L., Yang W. (2024). Pectin: A Review with Recent Advances in the Emerging Revolution and Multiscale Evaluation Approaches of Its Emulsifying Characteristics. Food Hydrocoll..

[7] Petkowicz C.L.O., Williams P.A. (2020). Pectins from Food Waste: Characterization and Functional Properties of a Pectin Extracted from Broccoli Stalk. Food Hydrocoll..

[8] Wang Z., Song W., Song H., Huang W., Li Y., Feng J. (2024). Effects of Extraction Methods on the Physicochemical Properties and Functionalities of Pectic Polysaccharides from Burdock (*Arctium lappa* L.). Int. J. Biol. Macromol..

[9] Muñoz-Almagro N., Valadez-Carmona L., Mendiola J.A., Ibáñez E., Villamiel M. (2019). Structural Characterisation of Pectin Obtained from Cacao Pod Husk. Comparison of Conventional and Subcritical Water Extraction. Carbohydr. Polym..

[10] Yang L., Zhang H., Zhao Y., Huang J., Zhu D., Wang S., Zhu L., Chen L., Xu X., Liu H. (2020). Chemical Structure, Chain Conformation and Rheological Properties of Pectic Polysaccharides from Soy Hulls. Int. J. Biol. Macromol..

[11] Kian L.K., Saba N., Jawaid M., Sultan M.T.H. (2019). A Review on Processing Techniques of Bast Fibers Nanocellulose and Its Polylactic Acid (PLA) Nanocomposites. Int. J. Biol. Macromol..

[12] Rehman A., Ahmad T., Aadil R.M., Spotti M.J., Bakry A.M., Khan I.M., Zhao L., Riaz T., Tong Q. (2019). Pectin Polymers as Wall Materials for the Nano-Encapsulation of Bioactive Compounds. Trends Food Sci. Technol..

[13] Shen P., Gao Z., Fang B., Rao J., Chen B. (2021). Ferreting out the Secrets of Industrial Hemp Protein as Emerging Functional Food Ingredients. Trends Food Sci. Technol..

[14] Sim Y.Y., Nyam K.L. (2021). *Hibiscus cannabinus* L. (Kenaf) Studies: Nutritional Composition, Phytochemistry, Pharmacology, and Potential Applications. Food Chem..

[15] FAO (2023). FAOSTAT: Production/Yield Quantities of Hemp/Kenaf/Ramie in World. http://www.fao.org/faostat/en/#home.

[16] Lyu P., Zhang Y., Wang X., Hurren C. (2021). Degumming Methods for Bast Fibers—A Mini Review. Ind. Crops Prod..

[17] Wang J., Liu Z., Li X., Liu G., Zhao J. (2023). Elucidating Structure of Pectin in Ramie Fiber to Customize Enzyme Cocktail for High-Efficiency Enzymatic Degumming. Carbohydr. Polym..

[18] Vignon M.R., Garcia-Jaldon C. (1996). Structural Features of the Pectic Polysaccharides Isolated from Retted Hemp Bast Fibres. Carbohydr. Res..

[19] Zheng Z., Wang J., Liu Y., Zhao X. (2022). Simultaneous Degumming and Extraction of a Nature Gum from Raw Hemp. J. Nat. Fibers.

[20] Alexopoulou E., Li D., Papatheohari Y., Siqi H., Scordia D., Testa G. (2015). How Kenaf (*Hibiscus cannabinus* L.) Can Achieve High Yields in Europe and China. Ind. Crops Prod..

[21] Mao K., Chen H., Qi H., Qiu Z., Zhang L., Zhou J. (2019). Visual Degumming Process of Ramie Fiber Using a Microbial Consortium RAMCD407. Cellulose.

[22] Cheng L., Duan S., Feng X., Zheng K., Yang Q., Liu Z., Peng Y. (2019). Optimization of Pectin Extraction from Ramie by Orthogonal Methodology and Its Partial Physicochemical Properties. Nanosci. Nanotechnol. Lett..

[23] Tang J., Qin X., Repo-Carrasco-Valencia R., Yang X., Deng Y., Hou C., Yang X. (2025). Physicochemical, Functional and Antioxidant Properties of Four Polysaccharides Sequentially Extracted from Jute (*Corchorus olitorius* L.) Leaves. Int. J. Biol. Macromol..

[24] Filisetti-Cozzi T.M.C.C., Carpita N.C. (1991). Measurement of Uronic Acids without Interference from Neutral Sugars. Anal. Biochem..

[25] Bradford M.M. (1976). A Rapid and Sensitive Method for the Quantitation of Microgram Quantities of Protein Utilizing the Principle of Protein-Dye Binding. Anal. Biochem..

[26] Kupina S., Fields C., Roman M.C., Brunelle S.L. (2018). Determination of Total Phenolic Content Using the Folin-C Assay: Single-Laboratory Validation, First Action 2017.13. J. AOAC Int..

[27] The National Academies Press (1981). Food Chemicals Codex.

[28] Wang C., Yu Y.-B., Chen T.-T., Wang Z.-W., Yan J.-K. (2020). Innovative Preparation, Physicochemical Characteristics and Functional Properties of Bioactive Polysaccharides from Fresh Okra (*Abelmoschus esculentus* (L.) Moench). Food Chem..

[29] Jiang Y., Xu Y., Li F., Li D., Huang Q. (2020). Pectin Extracted from Persimmon Peel: A Physicochemical Characterization and Emulsifying Properties Evaluation. Food Hydrocoll..

[30] Wang W., Ma X., Jiang P., Hu L., Zhi Z., Chen J., Ding T., Ye X., Liu D. (2016). Characterization of Pectin from Grapefruit Peel: A Comparison of Ultrasound-Assisted and Conventional Heating Extractions. Food Hydrocoll..

[31] Zhang S., Waterhouse G.I.N., Cui T., Sun-Waterhouse D., Wu P. (2023). Pectin Fractions Extracted Sequentially from Cerasus Humilis: Their Compositions, Structures, Functional Properties and Antioxidant Activities. Food Sci. Hum. Wellness.

[32] Bayar N., Bouallegue T., Achour M., Kriaa M., Bougatef A., Kammoun R. (2017). Ultrasonic Extraction of Pectin from Opuntia Ficus Indica Cladodes after Mucilage Removal: Optimization of Experimental Conditions and Evaluation of Chemical and Functional Properties. Food Chem..

[33] Zhao X., Zhou Y., Wu Z., Chen J., Zhou F., Zhao G. (2023). Thickening Effects of Ca2+ on Apple High-Methoxyl Pectin: Dependences on Ca2+ Concentration and the Degree of Esterification. Food Hydrocoll..

[34] Hosseini S.S., Khodaiyan F., Kazemi M., Najari Z. (2019). Optimization and Characterization of Pectin Extracted from Sour Orange Peel by Ultrasound Assisted Method. Int. J. Biol. Macromol..

[35] Muñoz-Almagro N., Ruiz-Torralba A., Méndez-Albiñana P., Guerra-Hernández E., García-Villanova B., Moreno R., Villamiel M., Montilla A. (2021). Berry Fruits as Source of Pectin: Conventional and Non-Conventional Extraction Techniques. Int. J. Biol. Macromol..

[36] Čopíková J., Taubner T., Tůma J., Synytsya A., Dušková D., Marounek M. (2015). Cholesterol and Fat Lowering with Hydrophobic Polysaccharide Derivatives. Carbohydr. Polym..

[37] Yang N., Wang D., Geng Y., Man J., Gao Y., Hang Y., Zheng H., Zhang M. (2022). Structure, Physicochemical Characterisation and Properties of Pectic Polysaccharide from Premma Puberula Pamp. Food Hydrocoll..

[38] Aritkhodzhaev K.A., Arifkhodzhaev A.O., Yusupov A.M. (1995). Pectin from Green Kenaf Bast. Chem. Nat. Compd..

[39] Pasandide B., Khodaiyan F., Mousavi Z.E., Hosseini S.S. (2017). Optimization of Aqueous Pectin Extraction from *Citrus medica* Peel. Carbohydr. Polym..

[40] Pakarinen A., Zhang J., Brock T., Maijala P., Viikari L. (2012). Enzymatic Accessibility of Fiber Hemp Is Enhanced by Enzymatic or Chemical Removal of Pectin. Bioresour. Technol..

[41] Wang H., Qiu C., Chen L., Abbasi A.M., Guo X., Liu R.H. (2019). Comparative Study of Phenolic Profiles, Antioxidant and Antiproliferative Activities in Different Vegetative Parts of Ramie (*Boehmeria nivea* L.). Molecules.

[42] Yap P.G., Gan C.Y. (2024). Optimized Extraction and Characterization of Ramie Leaf Polysaccharides Using Deep Eutectic Solvent and Microwave: Antioxidant, Metal Chelation, and UV Protection Properties. Int. J. Biol. Macromol..

[43] Pang Y., Peng Z., Ding K. (2024). An In-Depth Review: Unraveling the Extraction, Structure, Bio-Functionalities, Target Molecules, and Applications of Pectic Polysaccharides. Carbohydr. Polym..

[44] Guo H., Du Y., Gao H., Liao Y., Liu H., Wu D., Gan R., Gao H. (2024). Isolation, Purification, Degradation of Citrus Pectin and Correlation between Molecular Weight and Their Biological Properties. LWT.

[45] Muñoz-Almagro N., Vendrell-Calatayud M., Méndez-Albiñana P., Moreno R., Cano M.P., Villamiel M. (2021). Extraction Optimization and Structural Characterization of Pectin from Persimmon Fruit (Diospyros Kaki Thunb. Var. Rojo Brillante). Carbohydr. Polym..

[46] Lu J., Li J., Jin R., Li S., Yi J., Huang J. (2019). Extraction and Characterization of Pectin from Premna Microphylla Turcz Leaves. Int. J. Biol. Macromol..

[47] Cui J., Gu X., Wang F., Ouyang J., Wang J. (2015). Purification and Structural Characterization of an α-Glucosidase Inhibitory Polysaccharide from Apricot (*Armeniaca sibirica* L. Lam.) Pulp. Carbohydr. Polym..

[48] Reichembach L.H., Lúcia de Oliveira Petkowicz C. (2021). Pectins from Alternative Sources and Uses beyond Sweets and Jellies: An Overview. Food Hydrocoll..

[49] Liu J., Wang T., Huang B., Zhuang Y., Hu Y., Fei P. (2021). Pectin Modified with Phenolic Acids: Evaluation of Their Emulsification Properties, Antioxidation Activities, and Antibacterial Activities. Int. J. Biol. Macromol..

[50] Wan L., Chen Q., Huang M., Liu F., Pan S. (2019). Physiochemical, Rheological and Emulsifying Properties of Low Methoxyl Pectin Prepared by High Hydrostatic Pressure-Assisted Enzymatic, Conventional Enzymatic, and Alkaline de-Esterification: A Comparison Study. Food Hydrocoll..

[51] Kumar M., Tomar M., Saurabh V., Sasi M., Punia S., Potkule J., Maheshwari C., Changan S., Radha, Bhushan B. (2021). Delineating the Inherent Functional Descriptors and Biofunctionalities of Pectic Polysaccharides. Carbohydr. Polym..

[52] Asgari K., Labbafi M., Khodaiyan F., Kazemi M., Hosseini S.S. (2020). High-Methylated Pectin from Walnut Processing Wastes as a Potential Resource: Ultrasound Assisted Extraction and Physicochemical, Structural and Functional Analysis. Int. J. Biol. Macromol..

[53] Djaoud K., Muñoz-Almagro N., Benítez V., Martín-Cabrejas M.Á., Madani K., Boulekbache-Makhlouf L., Villamiel M. (2022). New Valorization Approach of Algerian Dates (*Phoenix dactylifera* L.) by Ultrasound Pectin Extraction: Physicochemical, Techno-Functional, Antioxidant and Antidiabetic Properties. Int. J. Biol. Macromol..

[54] Ahn S., Halake K., Lee J. (2017). Antioxidant and Ion-Induced Gelation Functions of Pectins Enabled by Polyphenol Conjugation. Int. J. Biol. Macromol..

[55] Siu K.-C., Chen X., Wu J.-Y. (2014). Constituents Actually Responsible for the Antioxidant Activities of Crude Polysaccharides Isolated from Mushrooms. J. Funct. Foods.

[56] Wang Z., Zheng Y., Lai Z., Hu X., Wang L., Wang X., Li Z., Gao M., Yang Y., Wang Q. (2024). Effect of Monosaccharide Composition and Proportion on the Bioactivity of Polysaccharides: A Review. Int. J. Biol. Macromol..

